# Transmission and evaporation of cough droplets in an elevator: Numerical simulations of some possible scenarios

**DOI:** 10.1063/5.0039559

**Published:** 2021-03-12

**Authors:** Nirvik Sen

**Affiliations:** Chemical Engineering Division, Bhabha Atomic Research Centre, Trombay, Mumbai 400085, India

## Abstract

As the world learns to live with COVID-19 and activities/business open up, the use of elevators becomes frequent. A pertinent question is what happens if someone accidentally coughs inside the elevator. In this work, a three dimensional Euler–Lagrangian model is used to understand the transmission and evaporation of micrometer-sized droplets in such cases. The effect of turbulence created by the air puff associated with coughing has been considered. Different possible scenarios varying in the presence of air ventilation within the elevator, number of persons coughing, direction of ejection of cough droplets, and ambient relative humidity and temperature have been postulated and simulated. The results obtained show that in the presence of proper ventilation within the elevator, most of the ejected cough droplets fall to the ground before impacting other persons traveling in the same elevator. However, in the absence of proper ventilation, the turbulence created during coughing transmits the particles all across the elevator enclosure.

## I. INTRODUCTION

The COVID-19 pandemic has impacted our lives in many ways. To keep COVID-19 at bay, all of us are faced with several dilemmas in our personal as well as professional lives (Bauernschmitt *et al.*, 2020; Chia and Oyeniran, 2020; Jaziri and Alnahdi, 2020; McCloskey *et al.*, 2020; Napoli *et al.*, 2020; Potter *et al.*, 2020; and Robert *et al.*, 2020). One of the dilemmas we face in day to day life is whether to use an elevator or use a staircase, if possible (Can I Get Coronavirus From Riding an Elevator?—The New York Times, 2020; Coronavirus Contamination of Apartment Buildings, 2020; New elevator etiquette, 2020; and Offices in COVID-19, 2020). The risk perceived in taking the elevator stems from the possibility of contamination through infected surfaces such as elevator buttons and hand rails. Risk is also perceived from the fact that it is very difficult to maintain social distancing in a small place such as an elevator. This dilemma has motivated researchers to use a numerical tool to understand and quantify the risk associated with using elevators. In this study, we numerically simulate more explicit risk scenarios, i.e., transmission and evaporation of micrometer-sized droplets generated due to coughing by one or more persons inside an elevator.

As cough droplets are ejected from the mouth of a person during coughing, transmission and evaporation of micrometer-sized droplets occur simultaneously. Under static conditions, the droplets should follow a projectile motion and tend to fall to the ground. At the same time, the water content inside the drop would also evaporate at a rate depending on relative humidity and temperature. However, in reality, the air puff generated due to coughing would create turbulence, which would be impacting the motion of the droplets. In fact, the turbulence created due to coughing tends to carry away finer droplets over significant distances (Li *et al.*, 2018). Significant research has been conducted to address the problem of transmission and evaporation of micrometer-sized droplets under different scenarios. Simulations to study transmission and evaporation of micrometer-sized droplets (due to coughing) inside a room having mixing or displacement type of ventilation were reported (Ji *et al.*, 2018). The authors did not consider the effect of insoluble components in cough droplets. Yang and co-workers used computational fluid dynamics (CFD) simulations to study the transmission and evaporation of micrometer-sized droplets inside a coach bus (Yang *et al.*, 2020). They considered the effect of insoluble components inside cough droplets. Spreading of cough droplets from one person to another under different ambient conditions was studied numerically (Feng *et al.*, 2020). The authors considered the effect of insoluble components inside cough droplets. Dbouk and Drikakis (2020a) reported a CFD model to study transmission and evaporation of droplets (due to coughing) in an open environment for different wind conditions and relative humidities. The authors did not consider the effect of insoluble components inside cough droplets. The effect of an inhomogeneous relative humidity field on the evaporation (as well as transmission) of cough droplets was reported (Li *et al.*, 2018). The authors considered the effect of insoluble components in cough droplets. The authors used a CFD model in their study. Yan and co-workers studied the thermal effect of the body of a person sitting in an otherwise quiescent environment in an enclosed space on the transmission and evaporation of micrometer-sized cough droplets (Yan *et al.*, 2019). They considered the effect of insoluble components in cough droplets. Recently, CFD simulations were used to numerically simulate how cough droplets transmit (while evaporating at the same time) in the presence of a face mask (Dbouk and Drikakis, 2020b). The authors did not consider the effect of insoluble components inside cough droplets. Recently, in their study on transmission of cough droplets in confined places such as a classroom, supermarket, and inside an elevator, Shao and co-workers reported a fraction of droplets deposited on the wall and a fraction vented out. However, they considered droplets emitting out of the mouth of a person during speaking and breathing (Shao *et al.*, 2020). They did not consider what happens when one coughs. The later scenario can be markedly different due to the presence of a turbulent air puff associated with coughing. These studies indicate that transmission and evaporation of droplets are affected by prevailing aerodynamics, temperature, and relative humidity. The studies also suggest that the equations describing the transmission and evaporation of such droplets are fairly well known. The authors did not comment on the treatment of insoluble components. Recently, the possibility of spread of virus laden particles due to urinal flushing was reported (Wang *et al.*, 2020). The authors integrated Volume of Fluids (VOF) and Discrete Phase Model (DPM) numerical techniques to study the toilet flushing process. They showed that the virus laden particles can rise upward and proposed that wearing masks be made mandatory inside washrooms. A CFD based technique with realistic modeling of a human sneeze was recently reported that was used to determine the spread of virus laden cough droplets (Busco *et al.*, 2020).

Studies pertaining to evaporation of cough/respiratory droplets from exposed surfaces have also been reported. Diffusion limited evaporation models have been used to estimate drying time for respiratory droplets (containing the virus) (Bhardwaj and Agarwal, 2020a). The role of surface wettability (contact angle) in the drying time of a virus laden respiratory droplet falling on a surface has also been reported (Bhardwaj and Agarwal, 2020b). Another such recent study reports the reason why the virus can survive on surfaces for a long time (days) (Bhardwaj and Agarwal, 2020c). Significant work has also been carried out to numerically determine how face masks/shields help reduce the chances of transmission and dispersion of virus laden respiratory/cough droplets (Akhtar *et al.*, 2020; Verma *et al.*, 2020a, b).

In this work, we developed a numerical technique to understand the transmission and evaporation of droplets generated due to coughing by one or more persons inside an elevator.

Different scenarios have been modeled to show the effect of ventilation in the elevator and what happens when more than one persons cough simultaneously. In addition, the effect of relative humidity and ambient temperature has also been studied. The model considers the dispersion of the cough droplets due to the turbulence created by the puff of air during coughing. As the temperature and relative humidity of the air puff gushing out of the mouth are different from the ambient conditions, the humidity/temperature field becomes inhomogeneous. The evaporation of the droplets as they move through the domain has been modeled. Finer details such as the effect of insoluble on evaporation dynamics have not been modeled as the thrust of the present work was to understand how cough droplets would spread through a relatively compact/cramped enclosure such as the interior of an elevator.

## II. NUMERICAL MODEL

### A. Geometry considered

**FIG. 1.**
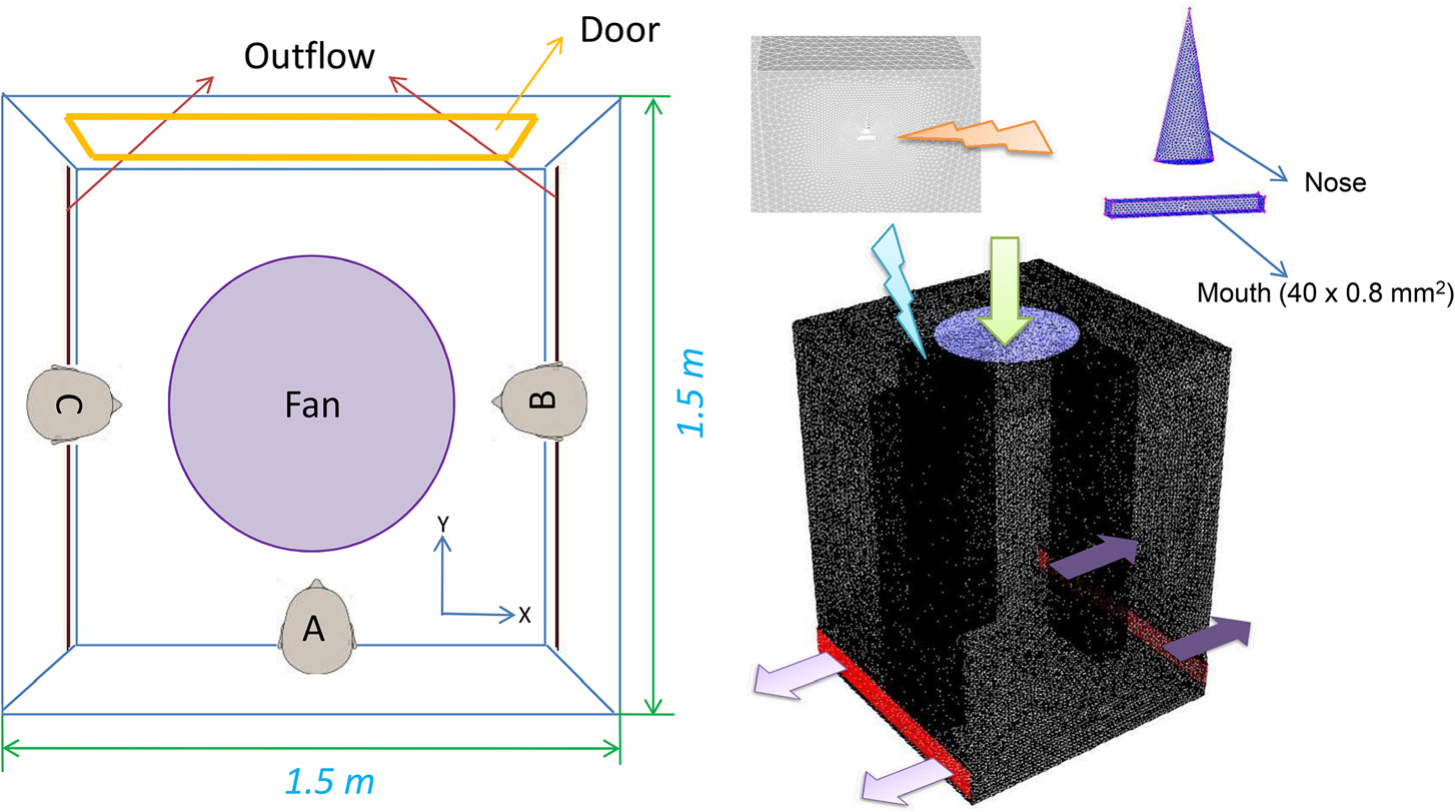
Top view of the computational domain (left) and meshed computational domain (right) (*human heads shown are only for representation*).

The computational domain is comprised of three persons inside an elevator. The dimension of the elevator considered is 1.5 × 1.5 × 2 m,^3^ which is a typical size of an elevator in a smaller residential building. A smaller size elevator is considered, as due to smaller space, the risk is expected to be more in a smaller elevator. The elevator is equipped with a top mounted fan (0.67 m diameter) which pushes down the air into the elevator. The air goes out from the ventilation slots provided at the bottom of side walls of the elevator. Three passengers standing along and in the middle of three walls are considered. The passengers are represented by rectangular boxes instead of exact three dimensional (3D) shapes to reduce modeling complexity as well as computational costs. The dimensions of the rectangle considered correspond to those of a representative human being in the standing posture. The height of each passenger is taken as 1.7 m (the location of the mouth being 1.6 m from bottom). The computational domain is shown in Fig. 1. Different scenarios have been simulated to understand the transmission and evaporation of droplets generated due to coughing by one or all of the fellow passengers. Micrometer-sized droplets generated due to the coughing of one/all of the passengers would travel in the domain depending on the prevailing velocity and associated turbulence field. A fraction of droplets might evaporate depending on temperature and humidity. A fraction may escape, while the rest will be deposited on various surfaces such as walls of the elevators and face/body of one of the persons traveling in the elevator.

### B. Governing equations

An Euler–Lagrangian model is used for numerical simulations. The flow of air is solved in an Eulerian frame of reference. Apart from the flow equations, energy and species transport equations are also solved to obtain the local variation in temperature and humidity fields due to the evaporation of droplets. This is important because evaporation and cooling of the droplets generated due to coughing would be strongly affected by the local temperature and humidity. Turbulence in air has been modeled by using the Reynolds Stress Model (RSM). A linear pressure strain correlation has been used. Cough droplets injected into the computational domain due to coughing by one or more persons are treated as discrete particles in a Lagrangian frame of reference. The droplets move around depending on the velocity field of the air and undergo evaporation while they move. At the instant of ejection from the mouth, the temperature of droplets as well as the moist air jet/puff (together comprising the droplet cloud) released from the mouth is considered to be equal to body temperature. However, as the cloud interacts with ambient air, it entrains a significant quantity of ambient air after which the cloud temperature would effectively be the same as ambient temperature (Agarwal and Bhardwaj, 2020). However, evaporation still continues (as long as the volatile component of the droplet exists), and the energy needed for the phase change to occur is extracted out of the droplet. This reduces the temperature of the droplet below the ambient/room temperature up to wet bulb temperature. Thus, eventually, droplets attain wet bulb temperature. The driving force for evaporation is the difference of water vapor partial pressure at the surface of the droplets and partial pressure of water vapor in the air surrounding the droplets. Apart from the driving force, the evaporation rate of water from the droplets will also depend on the mass transfer coefficient, which, in turn, will depend on the droplet Reynolds number. The droplets are considered to be pure water. Apart from mass transport, evaporative cooling was also modeled where energy transport from bulk air to the wet bulb temperature is simultaneously solved. The major forces acting on the droplets are the gravitational force, drag force, lift force, and virtual mass force. Due to the turbulence prevailing in the air phase, there would be additional dispersion of the droplets as they move in the domain. A random walk stochastic model has been used to account for this effect. This captures the interaction of the droplet with the instantaneous velocity components of turbulent flow of air. Equations solved for air and droplets are briefly summarized in Table I. A detailed explanation of the RSM governing equations can be found elsewhere (Parekh and Rzehak, 2018). A commercial finite volume solver has been used.

**TABLE I.** The governing equations solved for air and the droplets.

Governing equations for air	
Continuity equation	$\nabla \cdot \left(\vec{u}\right)=0$
Momentum equation	$\rho \frac{\partial \overset{\to }{u}}{\partial t}+\rho \left(\vec{u}\cdot \nabla \right)\vec{u}=\nabla .\left[-pI+\overset{=}{\tau }\right]+\rho_{c}\vec{g}$
Closure equation for the momentum equation	$\overset{=}{\tau }=\left(\overset{=}{\mu }+\mu_{T}\right)\left(\nabla \vec{u}+{\left(\nabla \vec{u}\right)}^{T}-\frac{2}{3}\left(\nabla \cdot \vec{u}\right)\overset{=}{I}\right)-\frac{2}{3}\rho k\overset{=}{I}$
Transport equation of the Reynolds stress tensor	$\rho \frac{\partial \overset{\equiv }{R}}{\partial t}+\nabla \cdot \left(\rho \vec{u}\overset{=}{R}\right)=\nabla .\left[\left(\overset{=}{\mu }+C_{s}{\overset{=}{\mu }}_{T}\right)\nabla \overset{=}{R}\right]+\rho \left(\overset{=}{P}+\overset{=}{\varphi }-0.67\varepsilon \overset{=}{I}\right)$
	*C*_*s*_ = 0.25
Transport equation of turbulent energy dissipation	$\rho \frac{\partial \varepsilon }{\partial t}+\nabla \cdot \left(\rho \vec{u}\varepsilon \right)=\nabla .\left[\left(\overset{=}{\mu }+C_{\varepsilon }{\overset{=}{\mu }}_{T}\right)\nabla \varepsilon \right]+\rho \frac{\varepsilon }{k}\left(C_{\varepsilon ,1}\frac{1}{2}tr\left(\overset{=}{P}\right)-C_{\varepsilon ,2}\right)$
	*C*_*ɛ*,1_ = 1.44; *C*_*ɛ*,2_ = 1.92
Reynolds stress tensor	$\overset{=}{R}=\left\langle {\vec{u}}^{\prime }{\vec{u}}^{\prime }\right\rangle$
Expression for turbulent kinetic energy	$k=0.5tr\left(\overset{=}{R}\right)$
Anisotropic turbulent viscosity	${\overset{=}{\mu }}_{T}=\rho \frac{k}{\varepsilon }\overset{=}{R}$
Turbulent production of Reynolds stress	$\overset{=}{P}=-\left(\left(\overset{=}{R}.\nabla \vec{u}\right)+{\left(\overset{=}{R}.\nabla \vec{u}\right)}^{T}\right)$
Energy balance	$\rho \frac{\partial H}{\partial t}+\nabla \cdot \left[\rho \vec{u}H-\nabla \left(k_{t}T\right)\right]=Qd$
Energy transport from bulk to droplet phase	$Qd=\overset{i=N}{\underset{i=1}{\sum }}\left(\pi d_{d,i}^{2}h\left(T-T_{d,i}\right)\right)$
	$\frac{hd_{d}}{k_{t}}=2.0+0.6Re_{d}^{0.5}Pr^{0.33}$
	$Pr=\frac{C_{p}\mu }{k_{t}}$
Species transport under turbulent flow conditions	$\rho \frac{\partial f_{v}}{\partial t}+\nabla \cdot \left[\rho \vec{u}f_{v}-\rho D_{eff}\nabla f_{v}\right]=Sv$
Closure term for the species transport equation	$D_{eff}=D_{mol}+\frac{\left|{\overset{=}{\mu }}_{T}\right|}{\rho Sc_{t}}$
	*Sc*_*t*_ = 0.7
Equation of motion of drop (identified by subscript d)	$m_{d}\frac{d\vec{u_{d}}}{dt}=\left(\rho_{d}-\rho \right)g+\frac{C_{D}\pi d_{d}^{2}\rho }{8}\left|\vec{u_{d}}\left.-\vec{u}\right|\left(\vec{u_{d}}-\vec{u}\right)\right.+F_{lift}+F_{vm}$
	$C_{d}=max\left\{\frac{24}{Re_{d}}\left(1+0.15Re_{d}^{0.687}\right);0.44\right\}$
	$F_{vm}=0.5\frac{\rho }{\rho_{d}}\frac{d}{dt}\left(\vec{u}-\vec{u_{d}}\right)$
	$F_{lift}=\frac{2Kv^{0.5}\rho d_{ij}}{\rho_{d}d_{d}{\left(d_{ik}d_{kl}\right)}^{0.25}}\left(\vec{u}-\vec{u_{d}}\right)$
Droplet evaporation term	$Sv=ak_{mt}\left(\frac{p_{sat}}{RT_{d}}-X\frac{p}{RT}\right)$
	$Nu=\frac{k_{mt}d_{d}}{D}=2.0+0.6Re_{d}^{0.5}Sc^{0.33}$
	$Re_{d}=\frac{d_{d}u_{p}\rho }{\mu }Sc=\frac{\mu }{\rho D}$
Conservation equation for droplet temperature	$m_{d}C_{p,d}\frac{dT_{d}}{dt}=ha\left(T-T_{d}\right)+\frac{dm_{d}}{dt}h_{fg}$

### C. Initial and boundary conditions

In Fig. 1, apart from the computational domain, the relevant boundary conditions are also shown. The fan is defined as the velocity inlet, while the two slots at the bottom of the elevator are defined as outflows. A time periodic velocity condition due to inhalation/exhalation has been defined at the face representing nose(s) of the person(s). All other surfaces, including the bodies of the passengers, are defined as the wall (with a no slip boundary condition). The mouth has been modeled as a rectangular slit of dimensions (40 × 0.8 mm^2^). Three different values of fan outflow velocities have been considered (0 m/s, 0.25 m/s, and 0.5 m/s). Exhalation of droplets from the mouth during coughing is accompanied with air gushing out of the mouth at a velocity of 8.5 m/s over a time duration of 0.12 s. The total mass of cough droplets is 7.7 mg. The droplets were injected across the entire mouth surface (40 × 0.8 mm^2^) as a distributed source. The droplets are assumed to have size distribution defined by Weibul's probability density function (mean droplet size of 80 *μ*m, n = 8). Size of the face representing mouth, initial velocity of cough droplets, and drop size distribution are as per a recent study (Dbouk and Drikakis, 2020a). For numerical simulations, the drop size distribution has been discretized into 10 classes. During exhalation, temperature and relative humidity of the puff of air ejecting out of the mouth are considered to be equal to body temperature and 100%, respectively.

## III. RESULTS AND DISCUSSION

### A. Validation of the numerical model

**FIG. 2.**
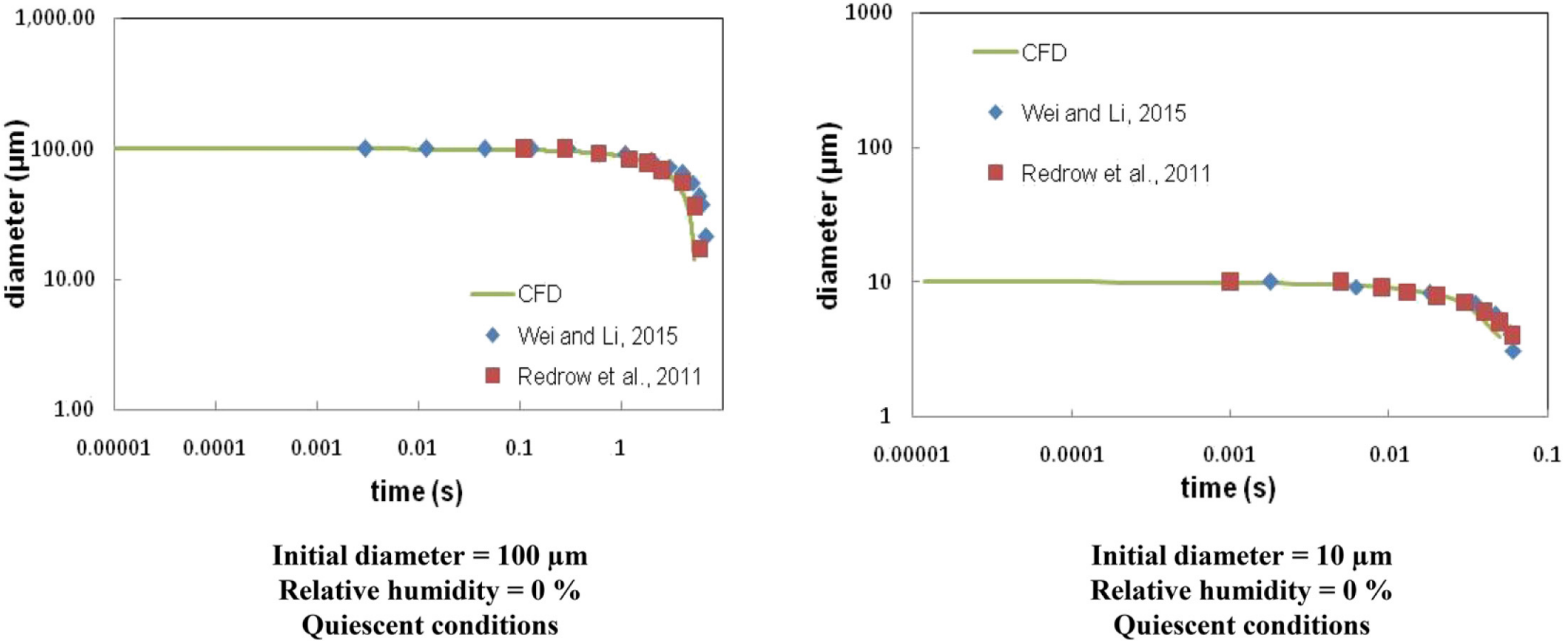
Validation of the CFD model with literature data.

The numerical model used in this study is validated with the reported data of change in diameter of an evaporating droplet with time under quiescent conditions (Redrow *et al.*, 2011; Wei and Li, 2015). The reported results are for two different initial drop diameters (10 *μ*m and 100 *μ*m) and at two different relative humidities (0% and 90%). The match between the reported and predicted variation of drop diameter for both cases is reasonably good. The geometry used for these validatory simulations is the same as shown in Fig. 1. However, to model the quiescent conditions, the computational domain is initialized with zero velocity (V_z_ = V_y_ = V_z_ = 0). Temperature is initialized to 25 °C throughout the domain, while two different uniform values of relative humidity (0% and 100%) are used. The cough is modeled by releasing a single particle stream (of same size, either 10 *μ*m or 100 *μ*m). The initial ejection velocity of the particle stream is kept fixed at 8.5 m/s. No turbulence is considered. The ejected particles follow a projectile motion, and as they fall toward the ground, their size reduces due to evaporation (Fig. 2).

### B. Grid independency test

**FIG. 3.**
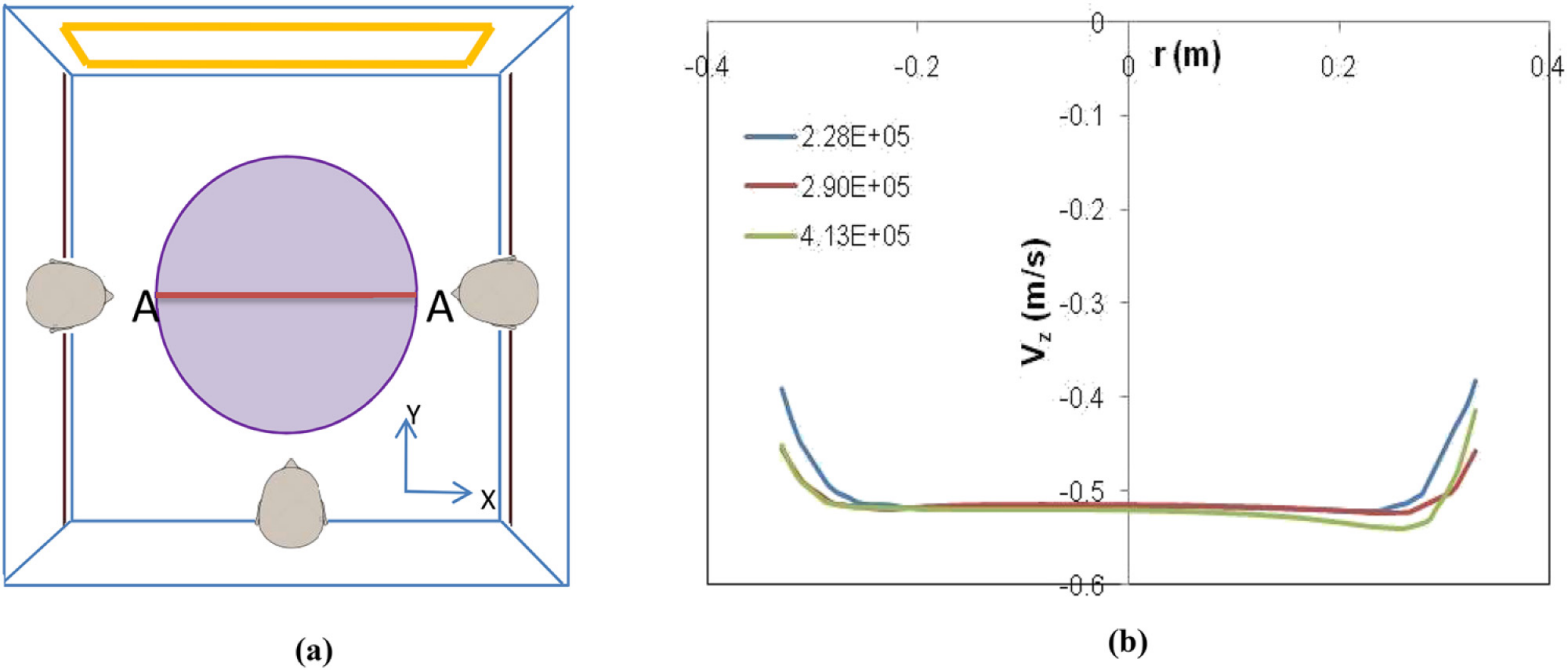
(a) Location of axial velocity (V_z_) tracking and (b) comparison of different grid densities on V_z_.

In this section, the effect of grid density on the flow field is determined. Unstructured (tetrahedral) mesh is used to discretize the computational domain. The effect of three different grid densities (2.28 × 10^5^, 2.90 × 10^5^, and 4.13 × 10^5^ cells/m^3^) on the flow field inside the elevator is investigated. The parameter tracked is the velocity (axial, V_z_) profile along a line midway across the elevator [line marked as AA in Fig. 3(a)]. The line AA is located at a height of 1 m from the elevator floor. Only Eulerian simulations are carried out. The fan speed is fixed at 0.5 m/s, while ambient temperature and relative humidity are held constant at 30 °C and 30%, respectively. The results are shown in Fig. 3(b). From Fig. 3(b), it can clearly be seen that a grid density of 2.90 × 10^5^ cells/m^3^ is sufficient to resolve the flow field inside the elevator. This grid density has been used throughout the work.

### C. Simulations for different scenarios

In this study, 11 different scenarios, as summarized in Table II, have been modeled. These are basically to understand the effect of fan speed, number of persons coughing, direction of coughing, ambient temperature, and relative humidity. The parameters tracked are the fraction (number) of droplets evaporated, fraction trapped on the elevator surface, fraction escaped, and fraction falling on other persons [except the person(s) coughing].

**TABLE II.** List of different scenarios simulated in this study.

	Objective	Fan speed (m/s)	Cough^a^ direction	Number of persons coughing	Temperature (°C)	Relative humidity (%)
Scenario 1	Effect of the fan speed	0.00	Normal	1	30	30
Scenario 2		0.25	Normal			
Scenario 3		0.50	Normal			
Scenario 4	Effect of the number of persons inside the elevator	0.25	Normal	2	30	30
Scenario 5			Normal	3		
Scenario 6	Effect of the direction of cough	0.00	30° to normal	1	30	30
Scenario 7			60° to normal			
Scenario 8	Effect of temperature	0.25	Normal	1	40	30
Scenario 9			Normal		50	
Scenario 10	Effect of relative humidity	0.25	Normal	1	30	50
Scenario 11			Normal			70

^a^ Normal direction means that the cough is aligned along the Y direction as shown in Fig. 1. In all cases, the direction of cough is horizontal (in the XY plane).

For all scenarios, except 4 and 5, the person who coughs is marked as “A” in Fig. 1. Scenarios 4 and 5 together with scenario 2 are aimed at understanding the effect of the number of persons coughing inside the elevator. Scenarios 6 and 7 along with scenario 2 are aimed at understanding what happens if the cough droplets are ejected at an angle to the normal. Scenarios 8 and 9 together with scenario 2 are aimed at understanding the effect of temperature. Scenarios 10 and 11 together with scenario 2 are aimed at understanding the effect of relative humidity. The ventilation system reported in this work is a once through ventilation system where the fresh air enters through the fan. This corresponds to non-air-conditioned elevators typical in countries such as India. Moreover, the choice of ambient temperature and relative humidity corresponds to a typical hot and humid day in a tropical country such as India.

### D. Scenarios 1–3

Table III shows the fate of the droplets for scenarios 1–3. In scenario 1 (fan off), if the person marked as “A” coughs, almost half of droplets evaporate before falling to the ground. The remaining fraction of droplets gets deposited onto the elevator surface. As will be seen subsequently, the droplets do not fall directly onto the floor but get entrained in the cough induced turbulence and spread across the elevator. However, if the fan is on and there is a stream of air directed toward the elevator floor, the fraction of cough droplets evaporating reduces and a major portion gets deposited onto the elevator surface (floor). In none of the scenarios 1–3, cough droplets exhaled by person “A” fall onto the other two persons (face).

**TABLE III.** Summary of the fate of the droplets exhaled in the coughing event for different levels of ventilation inside the elevator in scenarios 1–3.

	Fraction of droplets				
Scenario	Evaporated	Escaped	Trapped on the elevator surface	Trapped on person “B”	Trapped on person “C”
1 (No ventilation, person “A” coughs in a normal direction, RH = 30%, T = 30 C)	0.8932	0	0.1067	0	0
2 (V = 0.25 m/s, person “A” coughs in a normal direction, RH = 30%, T = 30 C)	0.75	0	0.25	0	0
3 (V = 0.5 m/s, person “A” coughs in a normal direction, RH = 30%, T = 30 C)	0.5046	0	0.495	0	0

**FIG. 4.**
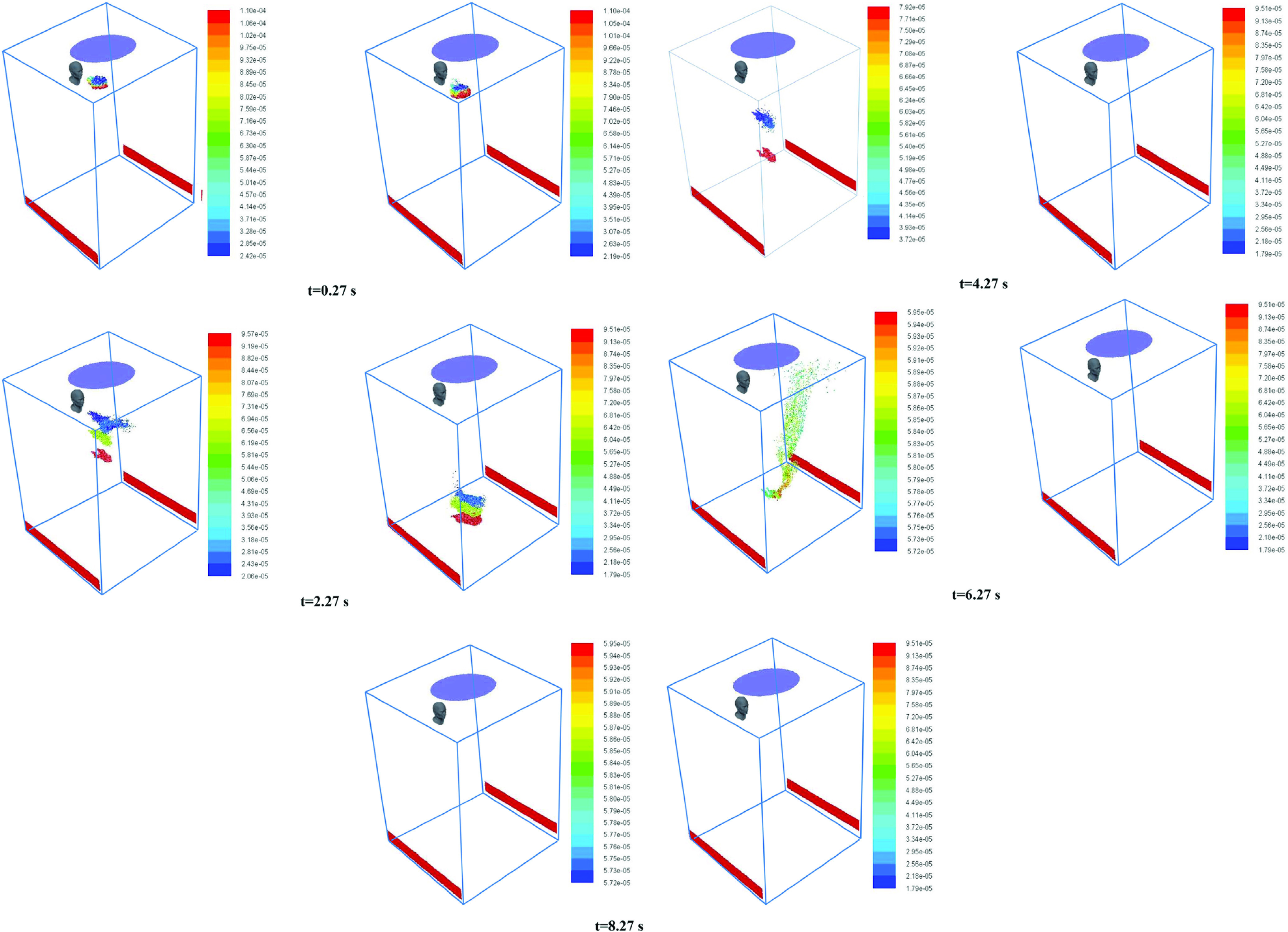
Snapshots of the cloud of droplets generated due to coughing by person “A” at different time instants at the start of the cough. The color scale is for the diameter of droplets. Figures on the left side are for scenario 1. Figures on the right side are for scenario 3 (*human heads shown are only for representation*).

Figure 4 shows the position of exhaled droplets in the elevator at different instants of times after exhalation. The figures on the left side are for scenario 1 (fan off), while those on the right side are for scenario 3 (fan velocity of 0.5 m/s). Under quiescent conditions, the only source of turbulence is the air that is exhaled along with the cough droplets that lasts only for a short duration of time (0.12 s). As they move, the droplets continue to evaporate. After ∼6 s, an interesting phenomenon is observed. Finer droplets (∼50 *μ*m) tend to move up and spread in the upper part of the elevator. This is attributed to the high velocity turbulent core of air released along with the cough. Even though it exists for a small duration of time, it continues to propagate as there is no other flow in quiescent conditions. The reason why this phenomenon happens after a time lag is because it takes some time for the droplets to undergo evaporation and reduce their size to the extent (∼50 *μ*m) that the turbulence generated due to the coughing process is effective in dragging them.

However, when the fan is on, the downward air flow drags the droplets toward the floor (essentially acting as an air curtain). Thus, a much larger fraction of droplets strike the floor. In this case also, a high velocity turbulent core of air is released when person A coughs. However, the continuous air flow from top suppresses this core, and it does not affect the movement of the droplets. Furthermore the drag force associated with air flow velocity of 0.5 m/s reduces the time for which the droplets remain airborne. This leads to a reduced fraction of droplets being evaporated when the fan is on. The faster movement of the cloud of droplets toward the floor for scenario 3 can be clearly observed from Fig. 4. Figure 4 also shows that the heavier particles, owing to their larger mass, tend to fall faster than the smaller lighter particles.

**FIG. 5.**
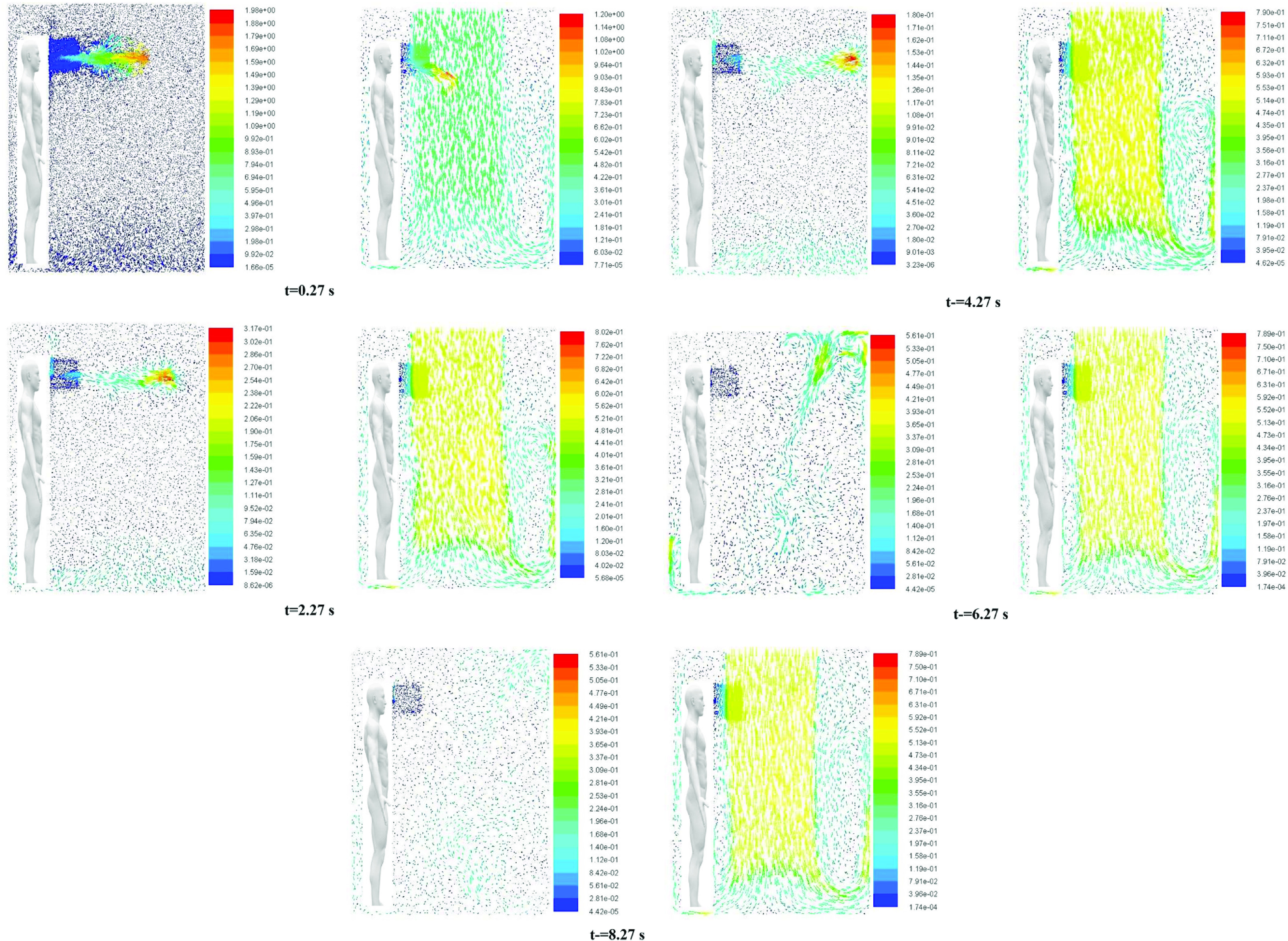
Velocity vectors in a longitudinal plane at different time instants after exhalation of cough droplets. Snapshots on the left side are for scenario 1. Snapshots on the right side are for scenario 3 (human figure shown are only for representation).

Figure 5 shows the velocity vectors in a longitudinal plane at the same time instants used in Fig. 4. The panel on the left shows the velocity vectors for the fan switched off, while that on the right shows the results for an air velocity of 0.5 m/s. It can be observed that for the case when the fan is off, the high velocity core generated due to coughing persists and the ensuing flow is directed upward after 6 s. It is this flow that carries the finer droplets toward the ceiling, as observed in Fig. 4. However, for the case of a fan velocity of 0.5 m/s, the downward air velocity toward the floor due to the fan does not allow the droplets to move up.

Hence, it can be concluded that the possibility of spread of fine droplets (∼50 *μ*m) is significant in the case of quiescent conditions. Thus, ventilation in terms of air flow from a top mounted fan and outflow from the lateral cutouts/slots located at the bottom is essential.

### E. Scenarios 4 and 5

**FIG. 6.**
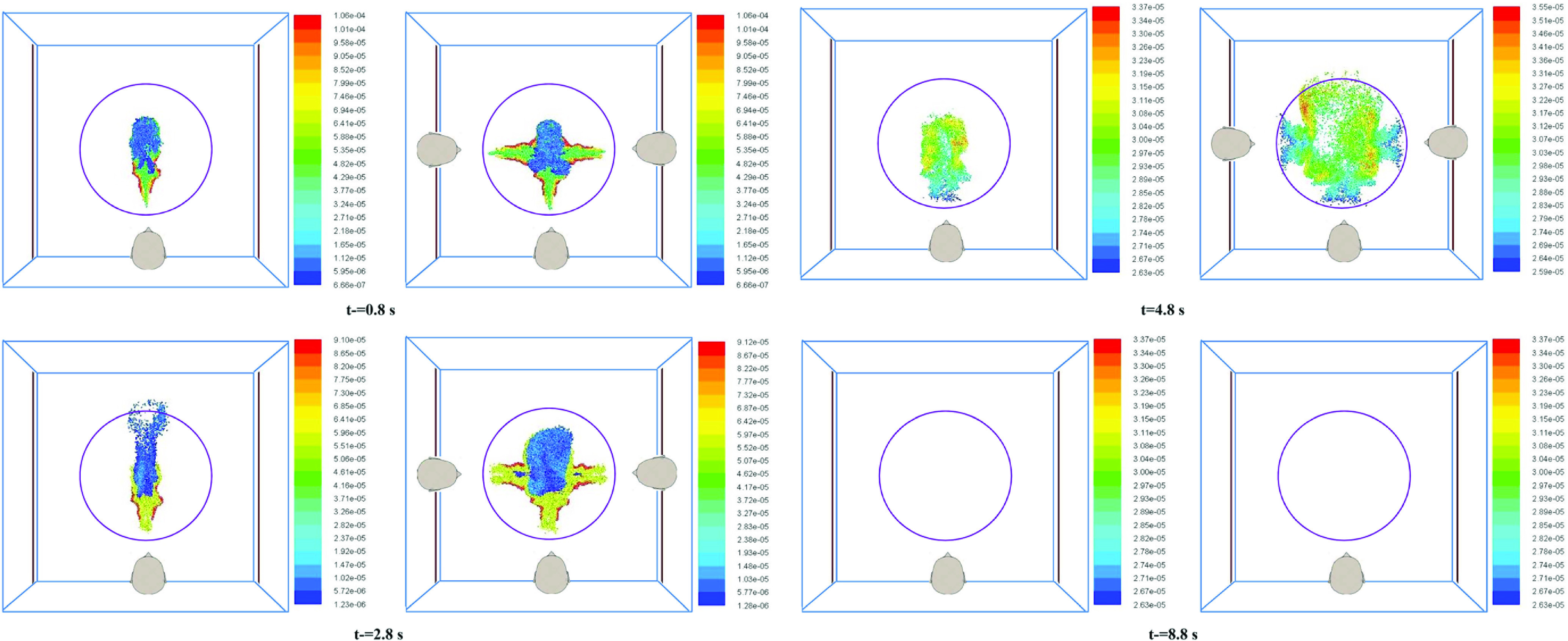
Snapshots of the droplet cloud exhaled due to coughing by a single person (left panel) and three persons simultaneously (right panel). The color scale is for drop diameter (*human head shown are only for representation*).

Table IV shows the fate of the droplets for scenarios 4 and 5 in Table II. For completeness, the results of scenario 2 are also included. In scenarios 2, 4, and 5, the number of persons who cough varies. The fan velocity is constant (0.25 m/s). Coughing by different persons (scenarios 4 and 5) occurs at the same instant of time to simulate the worst case, as in synchronized coughing, the number of droplets inside the elevator will be maximum. Table IV shows that a significant fraction of droplets evaporate for all three scenarios, and only a smaller fraction of droplets fall on the floor. However, there is no monotonic relation between the number of persons coughing and the fate of the droplets. Although the total number of droplets exhaled is proportional to the number of sources (persons coughing), there is not much difference in terms of the fraction of total droplets evaporated. This may be attributed to the similar nature of transmission and evaporation of the droplets as the passengers are placed symmetrically with respect to the central high velocity air core due to the fan at the top of the elevator. Figure 6 compares the cloud of exhaled droplets at three different instants of time for the case of one person coughing (scenario 1) and three persons coughing (scenario 5).

**TABLE IV.** Summary of the fate of the droplets exhaled in the coughing event for one, two, or three persons coughing simultaneously inside the elevator in scenarios 1, 4, and 5.

	Fraction of droplets				
Scenario	Evaporated	Escaped	Trapped on the elevator surface	Trapped on person “B”	Trapped on person “C”
2 (V = 0.25 m/s, person “A” coughs in a normal direction, RH = 30%, T = 30 C)	0.75	0	0.25	0	0
4 (V = 0.25 m/s, persons “A” and “B” cough in a normal direction, RH = 30%, T = 30 C)	0.8334	0	0.1665	0	0
5 (V = 0.25 m/s, persons “A,” “B,” and “C” cough in a normal direction, RH = 30%, T = 30 C)	0.7501	0	0.2498	0	0

Figure 6 shows that the trajectories of the droplets are similar, irrespective of whether one or three persons cough simultaneously. In the case of scenario 5, the droplets exhaled by three sources tend to collide at the center, beneath the top mounted fan. In the presence of a continuous air velocity from the fan, the droplets are dragged down and hit the floor within 5 s after being released. Even though the number density of droplets is more in the case when all three persons cough, the fraction of droplets evaporated remains similar because of the similar path droplets from each of the three sources take.

The results show that a centrally located fan provides an air curtain, which prevents droplets exhaled by one person during coughing from falling on the face of other persons. Although the case of distributed air supply is not simulated, it is expected that the best way of ventilation in an elevator is to have a centrally located top mounted fan rather than having multiple distributed sources of air supply (multiple fans). It may be noted that the outflow vents should preferably be located laterally close to the floor as has been considered in the present work. This ensures that the air stream hitting the ground can exit the elevator enclosure easily and in a way sweeps the entire elevator enclosure. In our opinion, such a ventilation scenario is better than the one where the entry and exit are located at the top (like the case modeled in Shao *et al.*, 2020) because in the latter case, there may be significant bypassing/short-circuiting of the incoming air stream.

### F. Scenarios 6 and 7

In previous scenarios shown until now, the persons are assumed to be standing symmetrically along the three walls of the elevator. The cough exhalation direction is in a horizontal plane perpendicular to the wall of the elevator. In reality, the cough exhalations may be in any arbitrary direction and in any arbitrary plane. The transmission and spread of droplets (especially deposition of droplets onto the face of other persons) may vary significantly in such scenarios. In this section, we simulate what happens when person “A” coughs at an angle to the normal direction (toward person “B”). The plane of coughing is however kept horizontal for comparison. As the most critical scenario is the case with no ventilation (scenario 1), for scenarios 6 and 7 also, quiescent conditions have been considered. Two different directions (30° and 60° with respect to the normal direction) have been considered. In the simulations pertaining to scenarios 6 and 7, the direction of the turbulent air puff and the release of the droplets have been considered at 30° and 60° to the normal. Table V shows the fate of the droplets generated due to coughing by person “A” for different directions of cough injections.

One thing that is clear from Table V is that the direction of cough plays a very critical role in determining whether other persons traveling in the elevator get affected. As person “A” coughs at an angle 30° with respect to the normal, a significant fraction of the cough droplets (∼40%) fall on the face of person “B.” For the case when the angle is 60° with the normal also, a small fraction of droplets fall onto the face of person “B.” This is in sharp contrast to the case where there was top down ventilation (scenarios 4 and 5). The results obtained in this section clearly demonstrate the risk of transmission of cough droplets from one person to another inside an elevator enclosure in case there is no ventilation inside the elevator.

**FIG. 7.**
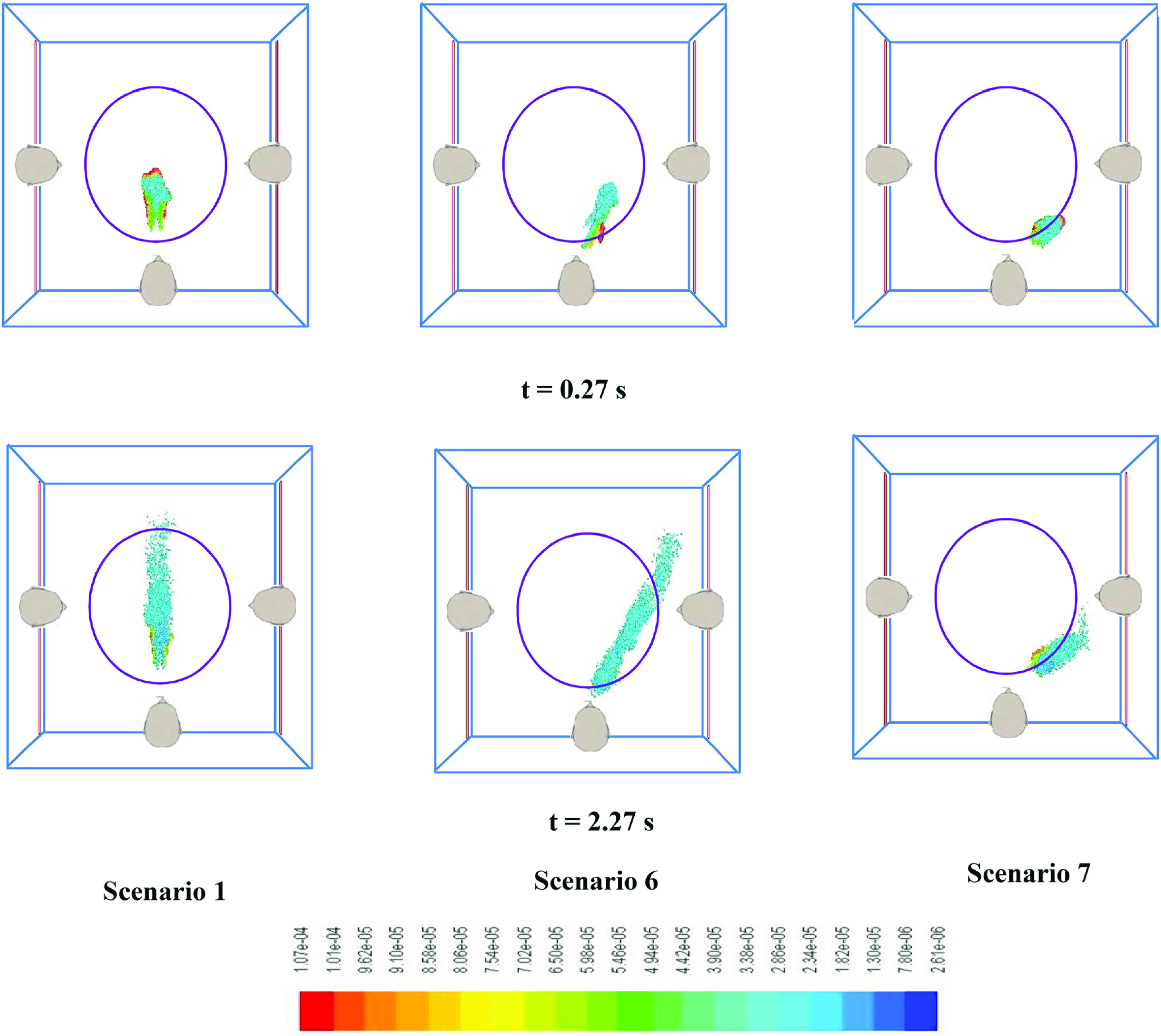
Snapshots of the droplet cloud exhaled due to coughing by a single person at three different angles. The color scale is for drop diameter (*human heads shown are only for representation*).

Figure 7 shows the comparison of spread and location of the cough droplets at two different instants of time (t = 0 corresponds to the instant of start of the coughing event) for all three scenarios (1, 6, and 7).

Figure 7 clearly shows that in case person “A” coughs at an angle to the normal, the cough droplets actually are propelled toward person “B.” In the present work, the cough direction is angled toward person “B.” Hence, person “B” is affected, while person “C” is not. It is reasonable to argue that in case the cough direction is toward person “C,” person “C” will be affected in a similar fashion. Hence, in the absence of ventilation, it can be concluded that there are significant chances of inter-personal transmission of cough droplets if one person coughs at an angle (directed toward other persons) inside an elevator.

### G. Scenarios 8 and 9

Table VI shows the fate of the droplets generated due to coughing by person “A” at different ambient temperatures. Simulations are carried out at ambient temperatures of 40 °C (scenario 8) and 50 °C (scenario 9). Scenario 2 in which temperature is 30 °C is also included for completeness. The choice of these temperatures corresponds to hot and humid conditions in tropical countries. Air flow velocity from the top mounted fan is kept constant at 0.25 m/s. Relative humidity is fixed at 30%. It can be observed that as the temperature increases, a greater fraction of droplets evaporate. This is anticipated as an increase in temperature increases the rate of mass loss due to evaporation. No fraction of droplets, however, falls onto the other persons.

**TABLE V.** Summary of the fate of the droplets exhaled during a coughing event at an angle to the normal direction in scenarios 1, 6, and 7.

	Fraction of droplets				
Scenario	Evaporated	Escaped	Trapped on the elevator surface	Trapped on person “B”	Trapped on person “C”
1 (No ventilation, person “A” coughs in a normal direction, RH = 30%, T = 30 C)	0.8932	0	0.1067	0	0
6 (No ventilation, person “A” coughs at 30° to the normal direction, RH = 30%, T = 30 C)	0.5856	0	0.0154	0.399	0
7 (No ventilation, person “A” coughs at 60° to the normal direction, RH = 30%, T = 30 C)	0.972	0	0.0278	0.0002	0

### H. Scenarios 10 and 11

Table VII shows the fate of the droplets generated due to coughing by person “A” at different relative humidity values. Air flow velocity from the top mounted fan is constant at 0.25 m/s. Temperature is kept constant at 30 °C. The simulations are carried out at relative humidities of 50% (scenario 10) and 70% (scenario 11). Once again, the choice of relative humidity is based on hot and humid conditions in tropical countries. The results for a relative humidity of 30% (scenario 1) are included for completeness. Table VII shows that as the relative humidity increases, the fraction of droplets evaporating decreases. This is attributed to a decrease in the driving force for evaporative mass loss at increased humidity. Thus, at a higher relative humidity, a greater fraction of the droplets fall on the floor. The probability of infection remains low as no droplets fall onto the other persons because of the presence of the continuous down draft of the air due to the top mounted fan.

**TABLE VI.** Summary of the fate of the droplets exhaled in the coughing event for different temperatures inside the elevator in scenarios 2, 8, and 9.

	Fraction of droplets				
Scenario	Evaporated	Escaped	Trapped on the elevator surface	Trapped on person “B”	Trapped on person “C”
2 (V = 0.25 m/s, person “A” coughs in a normal direction, RH = 30%, T = 30 C)	0.75	0	0.25	0	0
8 (V = 0.25 m/s, person “A” coughs in a normal direction, RH = 30%, T = 40 C)	0.75	0	0.25	0	0
9 (V = 0.25 m/s, person “A” coughs in a normal direction, RH = 30%, T = 50 C)	0.819	0	0.181	0	0

**TABLE VII.** Summary of the fate of the droplets exhaled in the coughing event for different relative humidities inside the elevator in scenarios 2, 10, and 11.

	Fraction of droplets				
Scenario	Evaporated	Escaped	Trapped on the elevator surface	Trapped on person “B”	Trapped on person “C”
2 (V = 0.25 m/s, person “A” coughs in a normal direction, RH = 30%, T = 30 C)	0.75	0	0.25	0	0
10 (V = 0.25 m/s, person “A” coughs in a normal direction, RH = 50%, T = 30 C)	0.5	0	0.5	0	0
11 (V = 0.25 m/s, person “A” coughs in a normal direction, RH = 70%, T = 30 C)	0.447	0	0.553	0	0

## IV. CONCLUSIONS

A 3D Euler–Lagrangian numerical model is used to simulate transmission and evaporation of micrometer-sized droplets generated due to coughing by one or more persons inside an elevator. An elevator typically used in multistoried residential buildings has been modeled. The elevator has a top mounted fan for air supply and outflow slots at the bottom of the side walls. As the droplets move according to the prevailing air flow pattern, they undergo evaporation depending on the temperature and relative humidity. Different scenarios have been simulated. The simulation results show that as long as the fan is switched on (air flow velocity 0.25 m/s–0.5 m/s), the droplets generated due to coughing are dragged down to the floor. However, if the fan is off, in the absence of a continuous down draft of the air, the cough droplets may circulate in the elevator due to the flow field generated by the air puff exhaled along with the cough droplets. Thus, the presence of a working fan is necessary to suppress this possible circulation of droplets. Results also show that in the presence of a continuous air stream due to the fan, the probability of cough droplets exhaled by a person falling on the other persons is much lower as long as the persons inside the elevator are standing symmetrically about the air stream generated by the top mounted fan. The results also suggest that it is better to have a centrally located fan instead of a distributed air supply. The problem associated with the fan being switched off intensifies in case a person coughs at an angle directed toward other persons in the elevator. In that case, there is significant chance (up to 40%) of cough droplets actually falling onto the face of other persons. The effect of temperature and relative humidity in the elevator enclosure was also studied. An increase in temperature and a reduction in relative humidity were seen to increase the fraction of droplets evaporating.

It may be noted that the scenarios simulated in this study are just a few of many possible real life scenarios in which the size of the elevator, air supply and outflow slots, position of a fan, and velocity may be different from what are considered in this study. The number of persons, their heights, and their relative positions inside the elevator may also vary. Some of these scenarios may pose significant risk in addition to the ones shown. Thus, it is advised to exercise due precautions while using an elevator.

## Data Availability

The data that support the findings of this study are available from the corresponding author upon reasonable request.

## NOMENCLATURE

*C*_*p*_: specific heat capacity of air (ML^2^/T^2^θ)
*C*_*p,d*_: specific heat capacity of droplets (ML^2^/T^2^θ)
*Cd*: drag coefficient
*d*_*d*_: droplet diameter (L)
*D*_*eff*_: effective diffusivity (L^2^/T)
*D*: molecular diffusivity in air (L^2^/T)
*F*_*vm*_: virtual mass force (ML/T^2^)
*F*_*elevator*_: elevator force (ML/T^2^)
*fv*: water vapor mass fraction
*f*: breathing frequency
*H*: enthalpy (L^2^/T^2^)
*h*: convective heat transfer coefficient (M/T^3^θ)
*h*_*fg*_: latent heat of vaporization (L^2^/T^2^)
*k*: turbulent kinetic energy (L^2^/T^2^)
*k*_*t*_: thermal conductivity (ML/T^3^θ)
*k*_*mt*_: mass transfer coefficient (for water vapor) (L/T)
*m*_*d*_: mass of droplets (M)
*p*: static pressure in the Navier–Strokes equation (M/LT^2^)
$\overset{=}{P}$: turbulent production of Reynolds stress (M/LT^3^)
$\overset{=}{R}$: Reynolds stress tensor (M/LT^2^)
*RH*: percent of relative humidity
*T*: temperature (θ)
*T*_*d*_: droplet temperature (θ)
$\vec{u}$: velocity vector in the Navier–Strokes equation (L/T)
$\vec{u_{d}}$: droplet velocity (L/T)
*V*: wind velocity (L/T)
*X*: mole fraction of water vapor

**Greek letters**
*ε*: turbulent kinetic energy dissipation rate (L/T^3^)
*μ*: viscosity (M/LT) critical evaluation of the results
*ρ*: density (M/L^3^)
